# Before calling it FMF: *MEFV* variants of uncertain significance in autoinflammatory disease

**DOI:** 10.1093/rheumatology/keag252

**Published:** 2026-05-14

**Authors:** Veysel Cam, Elif Cingoz, Hulya Ercan Emreol, Dilara Unal, Yagmur Bayindir, Emil Aliyev, Mehmet Orhan Erkan, Ozlem Necipoglu Banak, Hazel Delal Dara Kar, Ozge Basaran, Yelda Bilginer, Erdal Sag, Seza Ozen

**Affiliations:** Division of Rheumatology, Department of Paediatrics, Hacettepe University Faculty of Medicine, Ankara, Turkey; Department of Paediatrics, Hacettepe University Faculty of Medicine, Ankara, Turkey; Division of Rheumatology, Department of Paediatrics, Hacettepe University Faculty of Medicine, Ankara, Turkey; Division of Rheumatology, Department of Paediatrics, Hacettepe University Faculty of Medicine, Ankara, Turkey; Division of Rheumatology, Department of Paediatrics, Hacettepe University Faculty of Medicine, Ankara, Turkey; Division of Rheumatology, Department of Paediatrics, Hacettepe University Faculty of Medicine, Ankara, Turkey; Division of Rheumatology, Department of Paediatrics, Hacettepe University Faculty of Medicine, Ankara, Turkey; Division of Rheumatology, Department of Paediatrics, Hacettepe University Faculty of Medicine, Ankara, Turkey; Division of Rheumatology, Department of Paediatrics, Hacettepe University Faculty of Medicine, Ankara, Turkey; Division of Rheumatology, Department of Paediatrics, Hacettepe University Faculty of Medicine, Ankara, Turkey; Division of Rheumatology, Department of Paediatrics, Hacettepe University Faculty of Medicine, Ankara, Turkey; Division of Rheumatology, Department of Paediatrics, Hacettepe University Faculty of Medicine, Ankara, Turkey; Division of Rheumatology, Department of Paediatrics, Hacettepe University Faculty of Medicine, Ankara, Turkey

**Keywords:** FMF, *MEFV* gene, variants of uncertain significance

## Abstract

**Objectives:**

Familial Mediterranean fever (FMF) is an autoinflammatory disease characterized by recurrent febrile attacks and serositis, with a high prevalence and carrier frequency of *MEFV* variants in eastern Mediterranean populations. In this setting, interpretation of *MEFV* variants of uncertain significance (VUS) is challenging, and their clinical relevance remains controversial. We aimed to describe the clinical characteristics of patients carrying mono- or biallelic *MEFV* VUS and to compare them with patients harbouring biallelic pathogenic *MEFV* variants, including assessment of FMF and PFAPA classification according to Eurofever/PRINTO criteria.

**Methods:**

This retrospective study included paediatric patients with recurrent autoinflammatory manifestations who underwent *MEFV* genetic analysis and were receiving colchicine. Patients were stratified by *MEFV* genotype, and clinical features, attack characteristics, treatment profiles and classification status were compared.

**Results:**

Patients with *MEFV* VUS exhibited fewer classical FMF features, such as serositis-related chest pain and arthritis, but more frequent atypical manifestations, including diarrhoea, oral aphthae and lymphadenopathy, along with longer attack duration. Measures of disease burden, including age at onset and attack frequency, were similar between groups. FMF criteria were fulfilled by approximately half of patients with VUS.

**Conclusion:**

Patients with autoinflammatory disease carrying *MEFV* variants of uncertain significance may exhibit atypical clinical features. Alternative diagnoses should be considered, and further genetic evaluation may be required.

Rheumatology key messages

- The phenotype of patients with autoinflammatory disease carrying *MEFV* variants of uncertain significance differs from that of patients with classical FMF.
- The presence of atypical clinical features should prompt consideration of broader genetic evaluation.

## Introduction

Familial Mediterranean fever (FMF) is an autoinflammatory disease predominantly affecting populations of eastern Mediterranean descent and follows an autosomal recessive inheritance pattern [1]. Clinically, FMF is characterized by recurrent febrile attacks accompanied by serositis (including peritonitis and pleuritis) and arthritis, reflecting dysregulated innate immune activation [2]. The diagnosis of FMF is established on the basis of characteristic clinical features in conjunction with genetic analysis of the *MEFV* gene [3].

FMF shows a high prevalence and carrier frequency in eastern Mediterranean populations [4]. In Türkiye, the estimated disease prevalence is ∼1 in 500 individuals, while *MEFV* carrier rates have been reported to range between 10% and 15% in population-based studies [5–7]. This combination of high disease frequency and widespread heterozygosity increases the likelihood of identifying *MEFV* variants during genetic evaluation, thereby amplifying the importance of careful interpretation of genetic findings within an appropriate clinical context [8].

Given this epidemiological background, expert groups have proposed structured recommendations for the diagnosis and classification of FMF and other autoinflammatory diseases [9]. These recommendations distinguish between genetically confirmatory genotypes, typically defined by the presence of pathogenic variants in both alleles, and non-confirmatory genotypes, which include variants of uncertain significance [10]. This distinction aims to reduce overdiagnosis of FMF in regions with high carrier rates and to promote consideration of alternative autoinflammatory conditions when appropriate.

Within this framework, the Eurofever/PRINTO collaborative group developed classification criteria for autoinflammatory diseases, including FMF and periodic fever, aphthous stomatitis, pharyngitis and cervical adenitis (PFAPA), integrating both genotype-based stratification and clinical criteria into standardized classification sets [11]. This comprehensive approach provides a structured means of evaluating recurrent inflammatory syndromes in clinical practice.

However, despite the availability of such classification frameworks, patients carrying mono- or biallelic variants of uncertain significance (VUS) in the *MEFV* gene continue to be managed largely within an FMF-centred context in the literature and clinical practice [12, 13]. Labelling such patients primarily on the basis of *MEFV* variants may lead to attribution of symptoms to FMF, potentially resulting in an over-diagnosis of FMF and reduced consideration of extended genetic testing, including next-generation sequencing-based autoinflammatory panels.

In this study, we aimed to describe the clinical characteristics of patients evaluated at a tertiary referral centre who carried mono- or biallelic *MEFV* VUS, and to identify features that distinguish them from patients with biallelic pathogenic *MEFV* variants. In addition, we assessed whether these genotype-defined groups fulfilled FMF or PFAPA classification criteria according to the Eurofever/PRINTO framework, with the objective of clarifying phenotypic patterns and potential diagnostic overlap in this clinically relevant population.

## Method

### Study design and patient selection

This retrospective study was conducted at a single tertiary paediatric rheumatology referral centre. Patients evaluated at the outpatient clinic between December 2015 and December 2024 were screened for eligibility, provided that they had at least one follow-up visit within the preceding 6 months. In our centre all patients presenting with recurrent inflammation, initially undergo testing for the *MEFV* gene. Patients were included if they had recurrent inflammatory episodes suggesting an autoinflammatory disease phenotype, and had undergone *MEFV* gene analysis. Subsequently, colchicine was started to all these patients as a standard of care. Patients without available genetic testing or with incomplete clinical data were excluded.

### Genetic stratification and rationale for group selection

Patients were stratified according to their *MEFV* genotype. Patients carrying pathogenic *MEFV* variants in both alleles were classified as having biallelic pathogenic genotypes and were used as the reference group, as these genotypes represent a genetically confirmatory background for familial Mediterranean fever according to the Eurofever/PRINTO framework.

Variant classification was performed according to the American College of Medical Genetics and Genomics (ACMG) criteria, and interpretation of *MEFV* variants was supported using the Infevers database for autoinflammatory diseases. Patients carrying mono- or biallelic *MEFV* VUS, without any pathogenic variants, were classified as having mono- or biallelic VUS genotypes and constituted the study group. This group represents a clinically relevant population with autoinflammatory features frequently encountered in clinical practice but lacking definitive pathogenic *MEFV* variants.

### Clinical data collection

Clinical and demographic data were retrospectively extracted from electronic medical records. Variables included sex, age at symptom onset and attack characteristics. To assess baseline disease severity, annual attack frequency was defined as the number of attacks reported prior to the initiation of colchicine, while current disease activity under treatment was evaluated based on the number of attacks during the 6 months preceding the most recent follow-up visit. All variables were systematically collected for all patients at both baseline and follow-up time points. Clinical manifestations, including fever, abdominal pain, chest pain, arthritis, myalgia, lymphadenopathy, oral aphthae, diarrhoea and skin rash were recorded if present at the initial presentation or if they newly emerged during the follow-up period. Parental consanguinity status and use of anti-IL-1 therapy were also recorded.

### Phenotypic and classification assessment

Phenotypic characteristics and classification status were evaluated using the Eurofever/PRINTO clinical classification criteria, based on the predefined clinical items outlined, and not the criteria that includes genotype, of the original Eurofever/PRINTO classification study [11].

### Statistical analysis

Categorical variables were compared using the χ^2^ test or Fisher’s exact test, as appropriate. Continuous variables were assessed for normality and compared using the Mann–Whitney *U*-test. A two-sided *P*-value <0.05 was considered statistically significant. Statistical analyses were performed using IBM SPSS Statistics for Windows, Version 27.0 (IBM Corp., Armonk, NY, USA).

Ethical approval was obtained from the Hacettepe University Ethics Committee (SBA 25/697). Due to the retrospective nature of the study and the use of anonymized data, informed consent was not required.

## Results

A total of 273 patients were included in the analysis. Of these, 134 patients carried mono- or biallelic variants of uncertain significance (VUS) in the *MEFV* gene, while 139 patients had biallelic pathogenic *MEFV* variants (Table 1).

**Table 1:** Comparison of clinical features, attack characteristics and treatment profiles by *MEFV* genotype.

Clinical characteristic	Mono- or biallelic VUS genotypes	Biallelic pathogenic genotypes	*P*-value
*MEFV* genotype, *n* (%)	E148Q/−: 102 (76.1)	M694V/M680I: 36 (25.9)	
	E148Q/E148Q: 11 (8.2)	M694V/V726A: 35 (25.2)	
	R408Q/−: 7 (5.2)	M694V/M694V: 31 (22.3)	
		M680I/M680I: 13 (9.4)	
Others	14 (10.5)	24 (17.2)	
Total	134 (100.0)	139 (100.0)	
Fever, *n* (%)	119/134 (88.8)	126/139 (90.6)	0.76
Diarrhoea, *n* (%)	33/134 (24.6)	11/139 (7.9)	**<0.001**
Abdominal pain, *n* (%)	100/134 (74.6)	121/139 (87.1)	**0.014**
Chest pain (serositis-related), *n* (%)	20/134 (14.9)	81/139 (58.3)	**<0.001**
Arthritis, *n* (%)	23/134 (17.2)	55/139 (39.6)	**<0.001**
Myalgia, *n* (%)	46/134 (34.3)	48/139 (34.5)	1.00
Lymphadenopathy, *n* (%)	51/134 (38.1)	4/139 (2.9)	**<0.001**
Oral aphthae, *n* (%)	60/134 (44.8)	4/139 (2.9)	**<0.001**
Erysipelas-like erythema, *n* (%)	4/134 (3.0)	15/139 (10.8)	**0.022**
Attack duration, median (IQR), h	72 (72–102)	48 (36–72)	**<0.001**
Annual number of attacks, median (IQR)	12 (8–16)	12 (6–20)	0.20
Number of attacks in the last 6 months, median (IQR)	0 (0–2)	1 (0–3)	0.61
Age at symptom onset, median (IQR), months	36 (12–61)	30 (18–60)	0.93
Anti-IL-1 therapy use, *n* (%)	6/134 (4.5)	20/139 (14.4)	**0.012**
Parental consanguinity, *n* (%)	21/124 (16.9)	27/121 (22.3)	0.37

*P*-values shown in bold are considered statistically significant (*P* < 0.05). IQR: interquartile range; VUS: variants of uncertain significance.

Within the VUS group, the most frequent genotype was E148Q/−, observed in 102 patients (76.1%), followed by E148Q/E148Q in 11 patients (8.2%). Other VUS genotypes were less frequent (Supplementary Table S1).

In the biallelic pathogenic group, the most common genotypes were M694V/M680I, identified in 36 patients (25.9%), and M694V/V726A, observed in 35 patients (25.2%), followed by M694V/M694V in 31 patients (22.3%).

Fever was common in both groups and showed no significant difference between them. In contrast, classical FMF features, including abdominal pain, serositis-related chest pain, arthritis and erysipelas-like erythema, were observed less frequently in patients carrying *MEFV* VUS compared with those with biallelic pathogenic variants (Table 1). In contrast, several clinical features not typically associated with FMF, including diarrhoea, lymphadenopathy and oral aphthae, were more frequent in the VUS group, while myalgia occurred at similar rates in both groups (Table 1).

Attack duration was longer in patients with VUS genotypes, with a median of 72 h (interquartile range [IQR] 72–102) compared with the typical 48 h (IQR 36–72) in the biallelic pathogenic group (*P* < 0.001). The annual number of attacks was similar between groups, with a median of 12 attacks per year (IQR 8–16) in the VUS group and 12 attacks per year (IQR 6–20) in the pathogenic group. The number of attacks in the last 6 months was also comparable, with medians of 0 (IQR 0–2) and 1 (IQR 0–3), respectively.

Age at symptom onset was similar between groups, with a median of 36 months (IQR 12–61) in the VUS group and 30 months (IQR 18–60) in the biallelic pathogenic group. Parental consanguinity was reported in 21 of 124 patients (16.9%) in the VUS group and 27 of 121 patients (22.3%) in the pathogenic group, with no statistically significant difference. Anti-IL-1 therapy was used in patients with colchicine-resistant disease. All treatments were administered as continuous therapy and none were given on demand. In the biallelic pathogenic group, 20 patients received anti-IL-1 therapy, including 12 treated with anakinra and eight who were switched from anakinra to canakinumab. In the VUS group, six patients received anti-IL-1 therapy, all of whom were treated with anakinra. Overall, anti-IL-1 therapy was significantly more frequent in the biallelic pathogenic group than in the VUS group (14.4% *vs* 4.5%, *P* = 0.012).

**Figure 1:**
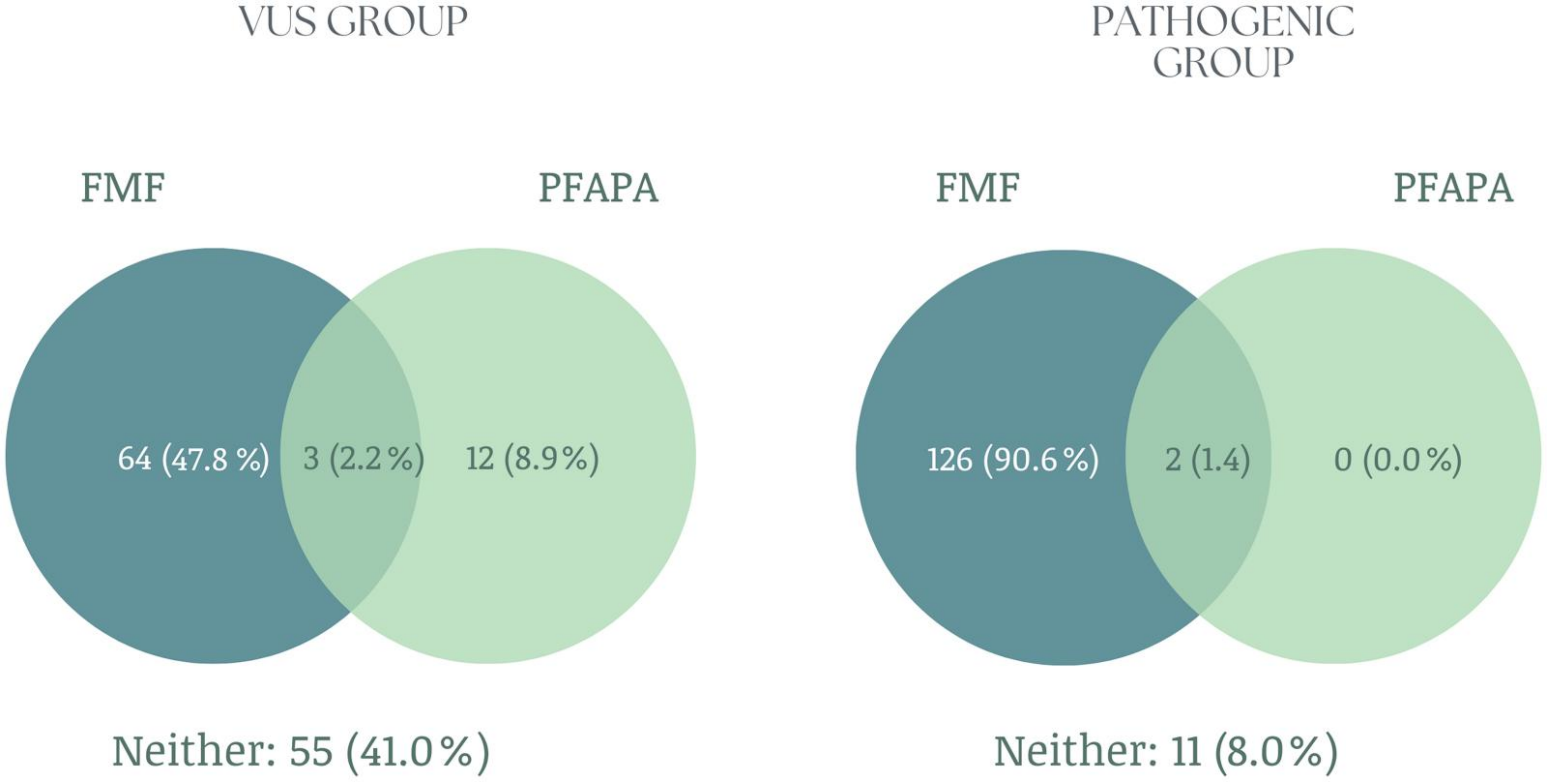
Clinical classification by *MEFV* genotype. Venn diagrams showing FMF and PFAPA phenotype distribution. Pathogenic variants are highly specific for FMF (90.6%), while VUS display clinical heterogeneity with a large proportion of unclassified cases (41.0%). FMF: familial Mediterranean fever; PFAPA: periodic fever, aphthous stomatitis, pharyngitis and cervical adenitis; VUS: variants of uncertain significance

According to the Eurofever/PRINTO classification criteria, 67 of 134 patients with *MEFV* variants of uncertain significance fulfilled FMF criteria, and a total of 79 patients were classified as having either FMF or PFAPA. In contrast, 55 patients (41%) in the VUS group did not fulfil either classification framework. In the biallelic pathogenic group, 126 of 139 patients (90.6%) fulfilled FMF classification criteria, a proportion similar to that reported in the original Eurofever/PRINTO cohort (Fig. 1).

## Discussion

In this study, we demonstrated that patients carrying mono- or biallelic *MEFV* variants of uncertain significance have a clinical profile distinct from those with biallelic pathogenic variants, characterized by a higher frequency of features not typically associated with FMF and longer attack duration. In contrast, parameters traditionally associated with disease severity were similar between groups.

These findings suggest that, particularly for E148Q, a heterozygous VUS may act as a non-specific inflammatory trigger (or confounding factor) for an unclassified autoinflammatory disease. Alternatively, other *MEFV* VUS may act as modifying SNPs within a broader, potentially polygenic inflammatory background [11]. Taken together, these results underscore the limitations of an FMF-centred diagnostic approach in patients with non-confirmatory *MEFV* genotypes and highlight the need for broader genetic evaluation.

The high prevalence of E148Q complicates its interpretation as a pathogenic variant and suggests low penetrance [6, 14, 15]. While several studies have associated E148Q and other VUS with a milder FMF phenotype, these analyses largely focused on patients with classical FMF features, and atypical manifestations were not systematically evaluated [12, 13, 16, 17]. In contrast, other studies have questioned the pathogenic role of isolated E148Q, with functional data showing cytokine profiles similar to healthy controls [18–21]. Additionally, E148Q has been identified in patients with undifferentiated autoinflammatory disease lacking classical FMF features, supporting a broader autoinflammatory context [22–24].

Following this overview of the E148Q-related discussion in the literature, returning to our own study, we aimed to move beyond a purely FMF-centred clinical framework. Rather than restricting inclusion to patients with classical FMF features, we deliberately included patients with autoinflammatory clinical presentations carrying E148Q, thereby adopting a broader approach than most previous studies. In this respect, our study differs from the existing literature, which has largely focused on FMF-consistent phenotypes when evaluating VUS genotypes.

Our findings suggest that atypical clinical features should be systematically evaluated in patients with *MEFV* VUS, particularly E148Q, rather than being interpreted solely within a classical FMF framework. While when together with a pathogenic variant E148Q may be associated with an atypical FMF phenotype, alternative autoinflammatory diagnoses should also be considered, and broader genetic evaluation, including next-generation sequencing (NGS), may be warranted in patients with atypical presentations.

This study has some limitations. In FMF-endemic countries such as Türkiye, *MEFV* testing is frequently performed during the evaluation of patients with recurrent inflammatory episodes. As a result, *MEFV* testing may be performed even in patients with clinical features compatible with PFAPA, and in patients with undifferentiated autoinflammatory disease, targeted *MEFV* testing may sometimes be performed before broader genetic testing such as NGS. In some cases, the detection of a single *MEFV* VUS may limit further investigation. Consequently, some patients referred to tertiary centres may have undergone *MEFV* testing because of suspected FMF, which introduces a potential risk of referral bias. Although this bias could theoretically increase the likelihood of including patients initially considered compatible with FMF, the clinical phenotype of the VUS group in our cohort remained clearly distinct from that of patients with biallelic pathogenic FMF variants. Nevertheless, this potential bias should be acknowledged as a limitation when interpreting our findings. It should also be noted that a large proportion of patients in the VUS group carried the E148Q variant (117/134). Given the relatively high carrier frequency of E148Q in Mediterranean populations and the ongoing debate regarding its pathogenic significance, the observed clinical patterns in this study may not reflect the effects of VUS genotypes as a whole. Accordingly, our findings should not be directly generalized to all *MEFV* VUS.

## Conclusion

Patients with autoinflammatory disease who carry *MEFV* variants of uncertain significance often present with clinical features atypical for FMF, and alternative diagnosis should be considered. Before classifying such patients as having FMF, consideration of PFAPA and additional clinical and genetic evaluation may be appropriate, particularly when atypical features are present. This approach may help avoid oversimplified classification and improve diagnostic accuracy in patients with non-confirmatory *MEFV* genotypes.

## Supplementary Material

keag252_Supplementary_Data

## Data Availability

The data used in this study are available from the corresponding author upon reasonable request.
