# Supplementary material for: Before calling it FMF: MEFV variants of uncertain significance in autoinflammatory disease
Source: Rheumatology (Oxford). 2026 May 14;65(6):keag252. doi: 10.1093/rheumatology/keag252 (PMC13244793; doi:10.1093/rheumatology/keag252)
Supplement: keag252_Supplementary_Data [file keag252_supplementary_data.docx]

**Supplementary Appendix**

**Supplementary Table S1. Distribution of MEFV genotypes in the study cohort**

| **MEFV genotype** | **VUS group n (%)** | **Pathogenic group n (%)** |
| --- | --- | --- |
| E148Q / – | 102 (76.1) | – |
| E148Q / E148Q | 11 (8.2) | – |
| R408Q / – | 7 (5.2) | – |
| E148Q / R408Q | 4 (3.0) | – |
| E148Q / K695R | 3 (2.2) | – |
| E167D / – | 3 (2.3) | – |
| K695R / – | 3 (2.3) | – |
| E148Q / V722M | 1 (0.7) | – |
| M694V / M680I | – | 36 (25.9) |
| M694V / V726A | – | 35 (25.2) |
| M694V / M694V | – | 31 (22.3) |
| M680I / V726A | – | 18 (13.0) |
| M680I / M680I | – | 13 (9.4) |
| V726A / V726A | – | 2 (1.4) |
| M680I / R761H | – | 2 (1.4) |
| M694V / R761H | – | 1 (0.7) |
| M694I / V726A | – | 1 (0.7) |
| **Total** | **134 (100)** | **139 (100)** |
